# Gene Profiling in Late Blight Resistance in Potato Genotype SD20

**DOI:** 10.3390/ijms19061728

**Published:** 2018-06-11

**Authors:** Xiaohui Yang, Xiao Guo, Yu Yang, Pei Ye, Xingyao Xiong, Jun Liu, Daofeng Dong, Guangcun Li

**Affiliations:** 1 Institute of Vegetables and Flowers, Shandong Academy of Agricultural Sciences, Molecular Biology Key Laboratory of Shandong Facility Vegetable, Jinan 250100, China; xiaohuiy_0601@163.com (X.Y.); guoxiaogzj@163.com (X.G.); yangyu97@163.com (Y.Y.); 2 Institute of Vegetables and Flowers, Chinese Academy of Agricultural Sciences, Key Laboratory of Biology and Genetic Improvement of Tuber and Root Crop, Ministry of Agriculture, Beijing 100081, China; xiongxingyao@caas.cn; 3 National Key Facility for Crop Resources and Genetic Improvement, Institute of Crop Science, Chinese Academy of Agricultural Sciences, Beijing 100081, China; 18829079611@snnu.edu.cn (P.Y.); liujun@caas.cn (J.L.)

**Keywords:** potato, late blight, *Phytophthora infestans*, transcriptome, resistance

## Abstract

Late blight caused by the oomycete fungus *Phytophthora infestans* (*Pi*) is the most serious obstacle to potato (*Solanum tuberosum*) production in the world. A super race isolate, CN152, which was identified from Sichuan Province, China, could overcome nearly all known late blight resistance genes and caused serious damage in China. The potato genotype SD20 was verified to be highly resistant to CN152; however, the molecular regulation network underlying late blight resistance pathway remains unclear in SD20. Here, we performed a time-course experiment to systematically profile the late blight resistance response genes using RNA-sequencing in SD20. We identified 3354 differentially expressed genes (DEGs), which mainly encoded transcription factors and protein kinases, and also included four NBS-LRR genes. The late blight responsive genes showed time-point-specific induction/repression. Multi-signaling pathways of salicylic acid, jasmonic acid, and ethylene signaling pathways involved in resistance and defense against *Pi* in SD20. Gene Ontology and KEGG analyses indicated that the DEGs were significantly enriched in metabolic process, protein serine/threonine kinase activity, and biosynthesis of secondary metabolites. Forty-three DEGs were involved in immune response, of which 19 were enriched in hypersensitive response reaction, which could play an important role in broad-spectrum resistance to *Pi* infection. Experimental verification confirmed the induced expression of the responsive genes in the late blight resistance signaling pathway, such as *WRKY*, *ERF*, *MAPK*, and NBS-LRR family genes. Our results provided valuable information for understanding late blight resistance mechanism of potato.

## 1. Introduction

The potato (*Solanum tuberosum* L.) is the third most important food crop in the world after wheat and rice, with an annual global production exceeding 374 million tons [1]. Potato production is highly affected by late blight, a disease caused by the oomycete *Phytophthora infestans* (*Pi*), which has become a serious obstacle to the development of the potato industry in China and around the world. Potato late blight caused the great famine of 1845–1852 in Ireland. During this famine around one million people died of hunger and one million more emigrated out of Ireland. Control of this disease is both expensive and time-consuming. The cost of control and damage by this disease is estimated at billions of U.S. dollars per year [2]. 

Since the Irish famine, potato late blight disease has attracted the attention of researchers and breeders, and breeding for resistance to the disease began after the famine. The most effective control method is to breed resistant varieties using the resistance derived from wild potato germplasms. So far, more than 20 late blight resistance (*R*) genes have been cloned including the broad-spectrum resistance genes *RB/Rpi-blb1*, *Rpi*-*blb2*, *Rpi-stol1* and *R8* [3,4,5,6], and the race-specific resistance genes *R1*, *R3a*, and *R3b* [7,8,9]. Functional stacking of *R*-genes has been employed to achieve resistance, and the cumulative effects of resistance to late blight have been monitored with three late blight resistance genes, *Rpi-sto1*, *Rpi-vnt1.1*, and *Rpi-blb3*, which were transferred into the susceptible potato cultivar Desiree [10]. Five resistance genes including four qualitative *R* genes *R3a*, *R3b*, *R4*, and *Rpi-Smira1,* and a quantitative *R* gene *Rpi-Smira2*, were stacked in Sárpo Mira, while the resistances were overcome by particular *Pi* strains [11]. Hence, an effective and robust control method relying on *R* genes is not feasible because the rapidly evolving pathogen breaks down these genes very quickly [12]. A previous study identified a potato late blight typical super race isolate CN152 from Sichuan strain of China, which could overcome all known late blight resistance genes including the broad-spectrum resistance *RB* gene [13]. Therefore, it is crucial to screen for late blight resistance potato germplasms and identify new *R* genes. In our previous resistance identification to over 20 distinguished potato accessions and genotype SD20, only SD20 was verified to be highly resistant to CN152 and showed typical hypersensitive response. However, the resistance genes and resistance mechanism remain uncovered in SD20.

Whole transcriptome shotgun sequencing, known as RNA-seq, is a widely used high-throughput approach that is known as second-generation sequencing technology. With the release of plant genome sequences including the potato [14], RNA-seq has becoming a powerful tool for transcriptome profiling, comparative gene expression analysis and gene identification [15,16,17]. Only several studies focused on using RNA-seq to identify genes involved in the potato response to late blight at the genome level. Using tetraploid cultivated potato Russet Burbank and its transgenic line SP2211 (+RB), RNA-seq was employed to perform transcriptome dynamics study of both potato tuber and foliage response to *Pi*, and the tubers of SP2211(+RB) showed increased transcription of defense related genes [18]. Further comparison of potato foliage-*Pi* with tuber-*Pi* interactions identified a set of differentially expressed genes and ontology groups that share components of the foliage and tuber response with *P. infestans* [19]. The transcriptomes of potato–pathogens interactions were compared among three potato clones and three wild *Solanum* species, and the results showed that resistant clones had more expressed putative *R*-genes than susceptible cultivar [20,21]. Here, we used a new potato genotype SD20 highly resistant to late blight and performed a time-course experiment to systematically profile the response genes to *Pi* infection using RNA-seq. The *Pi*-responsive genes and their expression patterns will help us to better explore key resistance genes to *Pi* and provide a molecular basis for plant–pathogen interactions.

## 2. Results

### 2.1. RNA-Sequencing and Transcriptome Assembly

**Figure 1:**
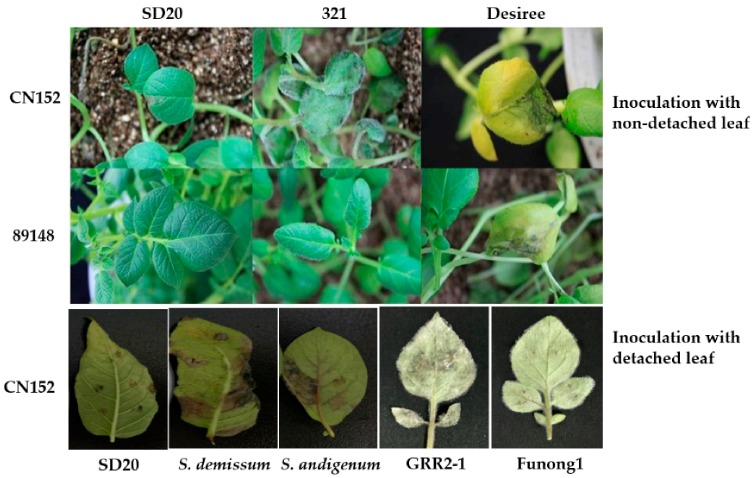
Inoculation with detached and non-detached leaves of different potato accessions using *Phytophthora infestans* isolates 89148 (race 0) and CN152 (race 1, 3b, 4, 5, 6, 7, 8, 9, 10, 11). SD20 and 321, tetraploid potato genotypes; Desiree, tetraploid potato cultivar as susceptible control; *S. demissum* and *S. andigenum*, wild potato species; GRR2-1, potato clone from *S. guerreroense*; Funong 1, a released variety in China.

**Figure 2:**
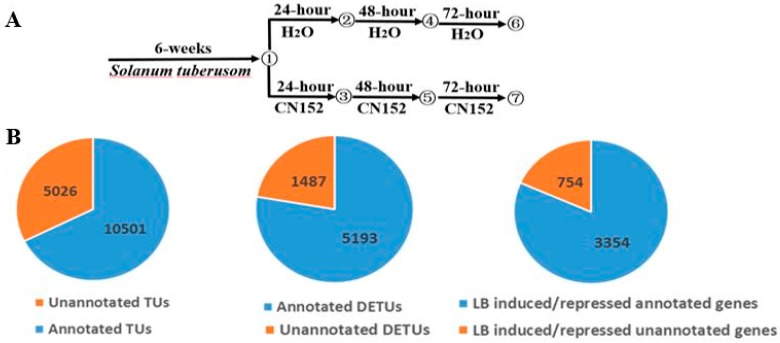
(**A**) A schematic showing the experimental design and the 16 sampling points. (**B**) Pie charts of the expressed TUs, differentially expressed TUs identified by pair-wise comparisons, and genes with expression levels altered in 24 h, 48 h, and/or 72 h samples relative to those in 0 h samples.

Through the identification of more than 20 potato accessions in our previous study, a late blight resistance tetraploid potato genotype SD20 was obtained (Figure 1). To gain a comprehensive knowledge of the transcriptional response of *S. tuberusom* to *Pi* infection, we conducted a transcriptome profiling analysis of SD20 that was inoculated with the *Pi* super race isolate CN152 for 0, 24, 48, and 72 h post infection (hpi) as well as H_2_O as the mock treatment (Figure 2A). Sixteen RNA libraries derived from seedling samples of 24, 48, and 72 hpi including untreated sample (0 h H_2_O) with two biological replicates were sequenced using the Illumina HiSeqX10 system with the 150-cycle paired-end sequencing protocol. 

An overview of the sequencing and mapping results is provided in Table 1. As shown, after data filtering and quality assessment, approximately 1263 million paired end reads were generated, yielding an average of 78.9 million paired end reads per sample. Subsequently, the clean high quality reads of all 16 libraries were processed by HISAT2 [22], Cufflinks [23], SAMtools [24], HTSeq [25], and DESeq2 [26]. Around 57.29–72.13% could be mapped uniquely to one location within the potato DM reference genome sequence. 

In total, we assembled and identified 27,436 expressed transcription units (TUs, normalized uniquely mapped reads count > 3 in at least one sample) in total. Among them, 10,501 TUs overlapped with annotated genes, while the other 5026 genes were suggested to be either unannotated or intergenic TUs (Figure 2B).

### 2.2. Differential Expression Analysis

We calculated and normalized fragments per kilobase of exon per million fragments mapped (FPKM) values for the assembled TUs based on the uniquely mapped reads. Using differential expression analysis of 16 pairwise comparisons with DESeq2 (fold-change of FPKMs > 2 between at least two samples, *p*-value < 0.05), we identified 10,991 total differentially expressed transcript units (DETUs) in response to the pathogen infection, including 5193 annotated DETUs and 1487 unannotated DETUs. Among all DEGs, there were 3354 annotated genes and 754 unannotated genes that were induced or repressed by late blight pathogen infection (Figure 2B).

According to the annotation, 3354 genes (DEGs) mainly encoded transcription factors (TFs), protein kinases (PKs), antioxidant enzymes, and pathogenesis-related (PR) proteins. TFs are one of the important regulators at the transcriptional level in higher plants. Here, 159 (4.7%) of all DEGs code for TFs from WRKY (11), MYB (18), ERF (11), bHLH (10), bZIP (five) and ZF (51) families. PKs act as signal transducer/receptor proteins in membranes and play a crucial role in phosphorylation events. A total of 174 (5.2%) PKs were found to be differentially regulated. These PKs were from families including serine/threonine protein kinase (STK), histidine kinase (HK), MAP kinase (MAPK), calcium-dependent protein kinase (CDPK), and receptor-like kinase (RLK). Nine RLKs were activated by *Pi* induction, including seven LRR-RLKs and two WAKs (wall-associated kinases). Moreover, 52 differentially expressed PRs (26 upregulated and 26 downregulated) were identified to contain PR1 (pathogenesis-related 1), glucan endo-1,3-β-d-glucosidase (PR-2), chitinase (PR-3), thaumatin (PR-5), proteinase inhibitor (PR-6) and non-specific lipid-transfer protein (PR-14). Among those, chitinase (PR-3) and b-1,3-glucanase (PR-2) are efficient in the lysis of chitin and glucan polymers of the fungal cell wall. 

In addition, four NBS-LRR coding genes (PGSC0003DMG400006800, PGSC0003DMG402004425, PGSC0003DMG400027797, and PGSC0003DMG400007999) were identified in this study. The gene PGSC0003DMG400007999 encoding *S. tuberosum* probable disease resistance protein At4g33300-like (ADR1) was differentially expressed at all three tested time points. The other three NBS-LRR coding genes were annotated as *S. tuberosum* putative late blight resistance protein homolog R1A-10, *S. tuberosum* putative disease resistance protein RGA3, and *S. tuberosum* TMV resistance protein N-like, respectively, and were only differentially expressed at 72 hpi. The expression profiles of the four NBS-LRRs genes are shown in Table 2.

Among the 3354 annotated DEGs, we also found a group of regulatory genes and marker genes associated with the salicylic acid (SA), jasmonic acid (JA), and ethylene (ET) signaling pathways (Table 2). Phenylalanine ammonialyase (PAL) is key enzyme, and *PR-1*, *PR-2* (*glucan endo-1,3-beta-glucosidase*), *NDR1* (*non-race-specific disease resistance 1*) are marker genes in SA signaling pathway. Here, we identified 4 *PAL* genes, 2 *PR-1* genes, 10 *PR-2* genes, and 1 *NDR1* gene in potato. All *PAL* and *PR-1* genes were up-regulated. Among the 10 *PR-2* genes, 6 ones were constantly up-regulated and 4 were down-regulated at the 3 time points. *NDR1* gene was only differentially up-regulated at 24 hpi. For JA signaling, LOX (lipoxygenase), AOS (allene oxide synthase), and PR-3 (chitinase) are key enzymes and the genes encoding these enzymes are considered as the marker genes of JA signaling pathway. Among the 3354 annotated DEGs, we found 4 up-regulated *LOX* genes, 1 up-regulated *AOS* gene and 5 up-regulated *PR-3* genes. The *ACC* (*1-Aminocyclopropane-1-carboxylic acid*) gene encoding the key enzyme of ET was also identified.

### 2.3. Clustering Analysis of Differential Expression TUs

**Figure 3:**
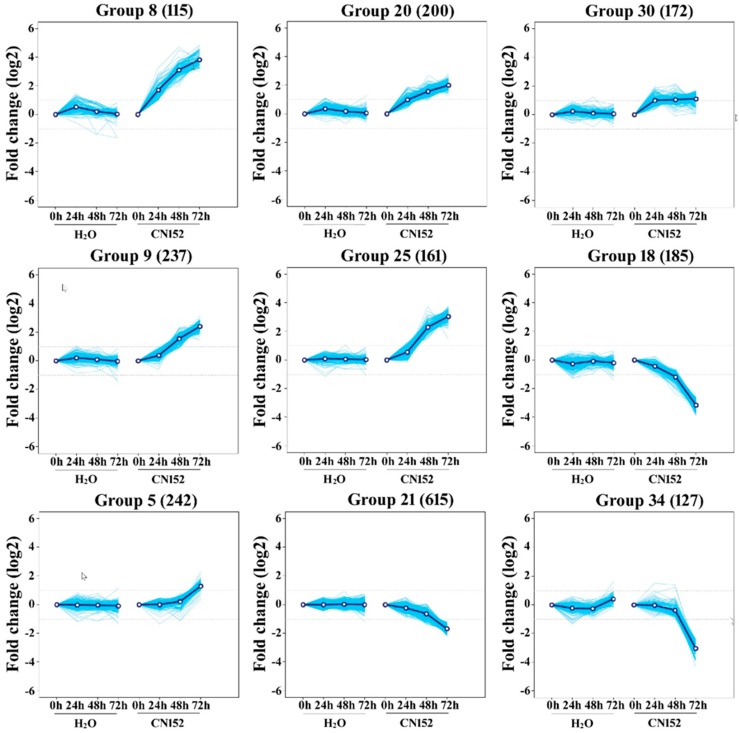
K-means clustering of the differential expression transcription units altered by *Phytophthora infestans* infection. Group 5, 8, 9, 20, 18, 21, 25, 30, and 34 are the nine representative clusters with different gene profiles; Numbers in the brackets are the number of genes for each group. *X*-axis, different time points of 0, 24, 48, and 72 h after CN152 inoculation and H_2_O treatment. *Y*-axis, log2 fold change of genes expression levels in 24, 48, and 72 hpi compared to 0 h; the dotted line means a value of 1.0, which indicates 2-fold greater expression in the cluster of interest.

Based on the differential expression levels, we classified all 6680 DETUs into 40 groups according to their relative expression profiles by performing K-means clustering analysis. We further classified the 6680 DETUs of these 40 groups into three major categories, according to whether the gene expression levels in 24, 48, and 72 hpi were 2-fold higher than at 0 h (Figure 3).

In the first category, the expression levels of around 854 DETUs were enhanced after inoculation and were 2-fold higher at 24 hpi compared with 0 h; moreover, the expression of around 500 genes was enhanced continuously with time extension of inoculation, which could be over 4-fold at 48–72 hpi compared with the control. In Figure 3, groups 8, 20, and 30 were classified into this category. The second category contained 1701 DETUs, including 1161 upregulated and 540 downregulated DETUs, which were constantly induced in response to time-course inoculation at 0, 24, 48, and 72 h. However, differences in gene expression were not 2-fold higher than the control until 48 h after inoculation. In Figure 3, groups 9, 18, and 25 were classified into this category. The third category included 4043 DETUs; differential expression did not reach over 2-fold change from control until 72 hpi. Only 1102 DETUs were upregulated and the other 2941 were downregulated. In Figure 3, groups 5, 21, and 34 were classified into this category.

### 2.4. Analysis of Annotated DEGs

According to the above clustering profiles, a total of 445, 898, and 1905 genes out of 3354 annotated DEGs were specifically induced at time points of 24, 48 and 72 hpi, respectively, when compared with these of control. These results indicated that these genes responded to the pathogen induction at a specific stage after inoculation. Among these genes, 422, 633, and 510 genes were upregulated, whereas 23, 265, and 1395 were downregulated at 24, 48, and 72 hpi, respectively. The number of DEGs, especially the number of downregulated ones, increased as time passed post inoculation, and the highest number of DEGs was found in 72 hpi. Moreover, 18, 45, and 86 transcription factors and 15, 47, and 110 protein kinases were detected at 24, 48, and 72 hpi, respectively. Among 3354 DEGs, 361 genes were found to be constantly differentially expressed at all three time points, with 343 gradually upregulated and 18 constantly downregulated genes. 

Among the 3354 DEGs, most showed 2- to 8-fold changes, while only a small portion of DEGs were greatly induced (more than 10-fold) by *Pi* inoculation. Six and seven genes were greatly upregulated more than 10-fold at 24 and 48 hpi, respectively; these genes encoded proteins including osmotin, glucan endo-1,3-beta-d-glucosidase, and divinyl ether synthase. Five and nine downregulated DEGs, coding for proteins including antifungal protein and SlTCP3, changed by 4-fold or more compared to control and were induced at 24 and 48 hpi, respectively. At 72 hpi, six upregulated (more than 6-fold) and eight downregulated (more than 8-fold) genes were greatly induced, respectively; these genes were annotated as xylem serine proteinase 1 and TIR-NBS-LRR disease resistance, for example. 

### 2.5. Gene Ontology Assignments of DEGs

**Figure 4:**
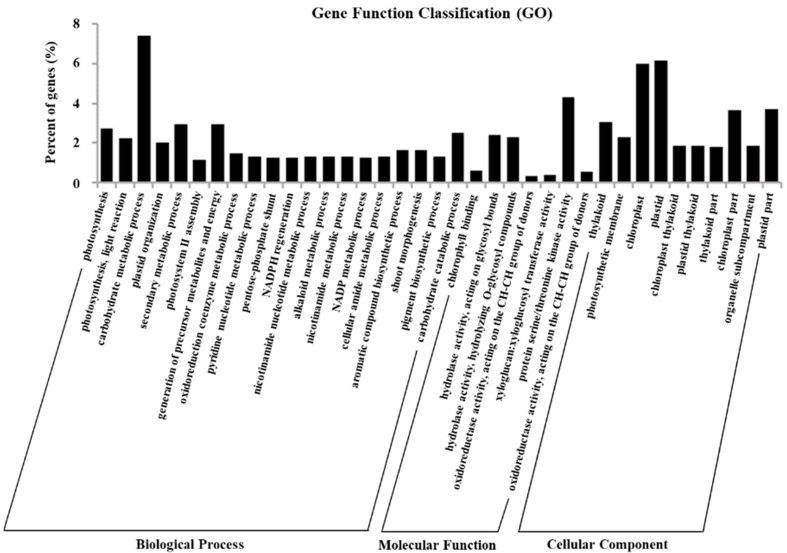
GO classification of annotated DEGs. *X*-axis, three major functional categories of GO terms: biological process, molecular function, and cellular component; *Y*-axis, terms with percentages of DEGs in the major category.

To get an overview of the function category of the genes that participated in the *Pi* infection response, the DEGs were subjected to Gene Ontology (GO) enrichment analysis. The 3354 DEGs fell into three main categories with corrected *p* < 0.05: a total of 205 terms were under biological process, 39 were under cellular component, and seven were enriched in molecular function. For the biological process category, metabolic process was the largest group, containing 79 terms (38.5%), including carbohydrate, secondary metabolites, oxidoreduction coenzyme, nucleotide, alkaloid, lipid, and amino acid metabolic/biosynthetic process. Among these terms, carbohydrate metabolic process had the most DEGs at 249. The second group was biosynthetic process (39 terms, 19.2%), followed by development, catabolic, morphogenesis, immune response, and localization processes, which included 1–11 GO terms, respectively. The top 10–20 enriched terms are in Figure 4.

**Figure 5:**
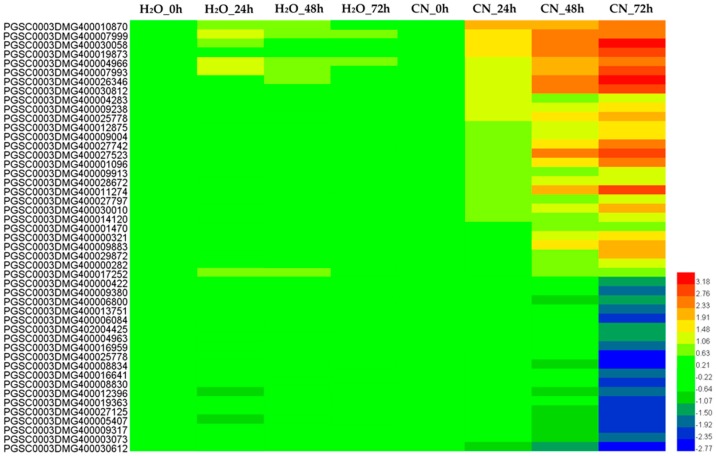
The expression fold-change of genes involved in immune response and HR actions. CN means *Phytophthora infestans* super isolate CN152. Gene expression levels in CN-0 h samples are given as controls.

Remarkably, six GO terms of immune response and two GO terms of plant-type hypersensitive response (GO:0009626 and GO:0010363) were highly enriched within the biological process category. Forty-three DEGs were involved in immune response, of which 19 were enriched in HR reactions with 10 upregulated and nine downregulated genes encoding for phytoalexin-deficient 4-2 protein (PAD4), MAPK, MAP kinase substrate 1 (MKS1), receptor protein kinase, and Ornithine aminotransferase (OAT) (Figure 5). These genes could play an important role in the broad-spectrum resistance to *Pi* infection in potato genotype SD20. DEGs involved in the immune response and HR actions with fold changes of their FPKM ratio (log2FC) between the inoculations and control are shown in Supplementary Table S1.

In the molecular function category, the 7 significantly enriched GO terms were involved in chlorophyll binding, hydrolase activity, oxidoreductase activity, xyloglucosyl transferase activity, and protein serine/threonine kinase activity (Figure 4). Most of the 144 DEGs were significantly enriched in protein serine/threonine kinase activity (GO:0004674) in this category. There were 39 significantly enriched GO terms in the cellular component category, which were mainly related to cell membrane (11 terms, 28.2%), cell organ (seven terms, 17.9%) and cell part (five terms, 12.8%). The terms “thylakoid,” “photosynthetic membrane,” “chloroplast,” “plastid,” and “chloroplast thylakoid” were among the top five ranks in the cellular component category (Figure 4). The membrane term (GO:0016020) had the most DEGs at 516.

### 2.6. Significantly Enriched KEGG Pathways in DEGs

**Figure 6:**
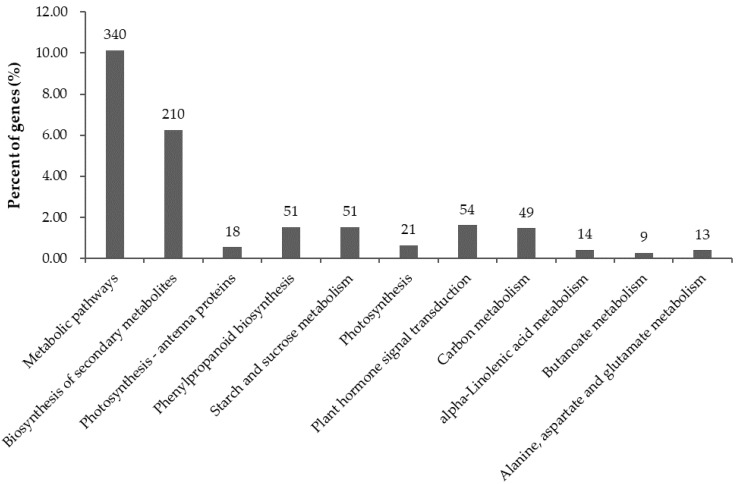
The distribution of pathways of differentially expressed genes (DEGs) annotated in the Kyoto Encyclopedia of Genes and Genomes (KEGG) data library. *X*-axis, the name of 11 significant pathways in KEGG; *Y*-axis, percentage of DEGs annotated in the pathway.

KEGG serves as a basic platform for systematic analysis of gene function in terms of gene product networks. To further identify biosynthetic pathways that are active in potato SD20 infected by CN152, the 3354 annotated DEGs were mapped to 113 reference canonical pathways. Twenty-eight canonical reference pathways were significant with *p*-value < 0.05, including 11 very significant pathways with *p*-value < 0.01 (Figure 6). The top two enriched pathways were metabolic pathways and biosynthesis of secondary metabolites with 340 and 210 DEGs. The other pathways contained around 6–51 DEGs. 

### 2.7. Verification of RNA-Seq Data Using Quantitative Real-Time PCR

**Figure 7:**
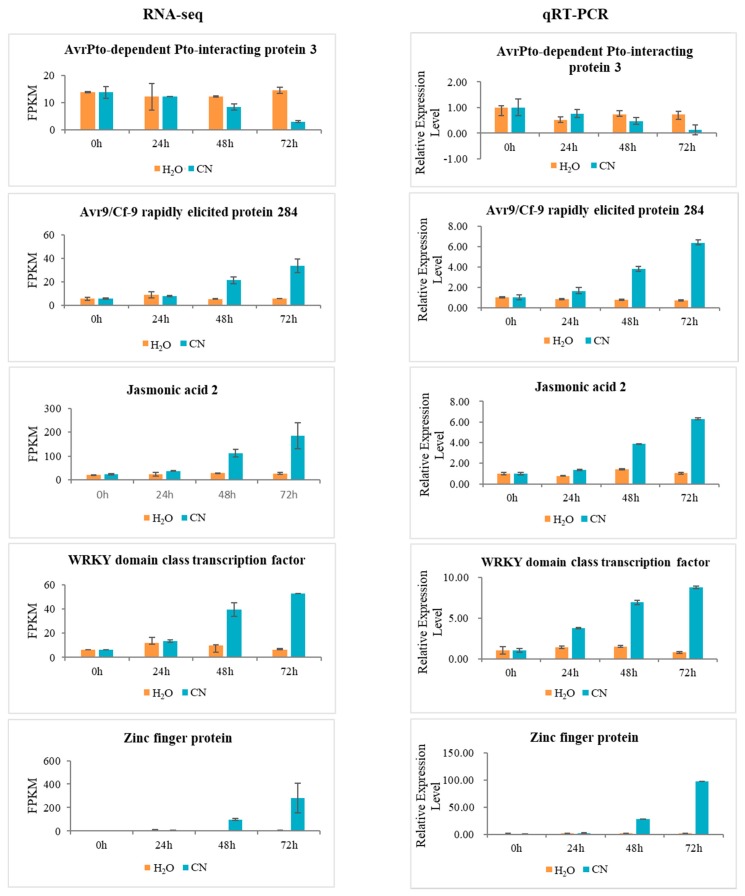

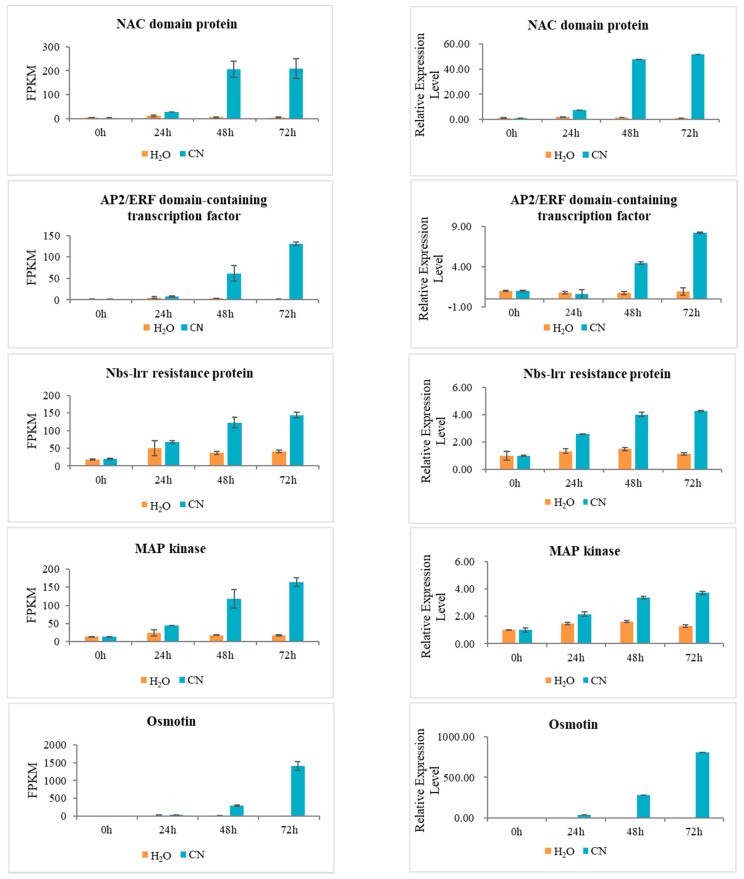
Gene expression profiles of RNA-seq and experimental verification of gene expression levels by qRT-PCR.

To validate the results of RNA-seq data, the expression levels of 10 randomly selected DEGs were analyzed by qRT-PCR. The results were in agreement with the observed changes in transcript abundance that were determined by RNA-seq analysis, which suggested that our transcriptome profiling data were highly reliable (Figure 7).

## 3. Discussion

### 3.1. Tetraploid Potato Genotype SD20 Is an Ideal Antigen for Late Blight

Late blight caused by *P. infestans* is the most devastating disease affecting potato production. This disease is hard to control because the *Pi* races possess high evolutionary potential, and can overcome known resistance genes. Recently, farmers have controlled late blight primarily with chemicals, but the chemical sprays are expensive and result in environmental pollution. In the present study, we found that an ideal potato antigen, genotype SD20, conferred high resistance to *Pi* super race isolate CN152 (race 1, 3b, 4, 5, 6, 7, 8, 9, 10, 11), which could overcome all known late blight resistance genes including *RB* gene. This genotype conferred a typical hypersensitive response when inoculated with CN152 using both detached and non-detached leaflets, indicating that the genotype SD20 may contain multiple *R* genes and/or broad-spectrum *R* genes, and probably possesses permanent resistance to late blight.

### 3.2. RNA-Seq and Differentially Expressed Genes (DEGs) Analysis

To detect resistance genes and resistance mechanisms in SD20, we performed a time-course analysis based on transcriptome profiling after *Pi* induction. After sequencing, we obtained an average of 11.8 G clean reads for each sample covering 16-fold of the potato haplotype DM reference genome (727 M). Eventually, we identified 27,436 differentially expressed transcription units (DETUs) in total, including 5026 encoding previously unannotated genes, which could be new protein coding genes. After analyzing DEGs, we detected 6680 DETUs responding to *Pi* induction, of which, 3354 were identified as annotated DEGs, and subsequently classified them into three categories according to their differential expression levels. Finally, we selected 10 representative DEGs to validate our gene annotations. qRT-PCR analysis showed expression patterns consistent with RNA-seq analysis. We are confident that our transcriptome database is a valuable addition to the publicly available autotetraploid potato genomic information.

EST and microarray approaches have often been used to identify defense-induced genes expressed in response to late blight [27]. However, few studies have focused on using RNA-seq to identify genes involved in the potato response to late blight at the genome level [18,19,20,21]. Using RNA-seq, 1680 DEGs were induced by late blight in potato haploid DM [28], and 2531 DEGs were generated in response to *Pi* inoculation in tubers of tetraploid potato and its transgenic *RB* lines [18]. In contrast, up to 3354 DEGs were obtained in this study, suggesting that there are many resistance-related genes in the genotype SD20 responding to the late blight super race CN152.

### 3.3. Resistance Gene Expression Profiling in Potato Genotype SD20

Plants use many methods of recognizing and defending against pathogens [29]. Plants recognize pathogen-associated molecular patterns (PAMPs) at the beginning of an infection, initiating PAMP-triggered immunity (PTI). The second line of defense in plants involves producing resistance proteins encoded by *R* genes that are specific for effectors produced by pathogens. This qualitative resistance is referred to as effector-triggered immunity (ETI) [30]. 

Induced defenses take many forms during the early stages of infection in plants: phytoalexins are synthesized; reactive oxygen species (ROS) are produced; cell walls are fortified; and the cytoskeleton is reorganized [31]. In later stages of infection, plants limit pathogen spread by transcribing pathogenesis-related (PR) proteins and developing the hypersensitive response (HR), which is a type of programmed cell death (PCD) [32]. In our previous study, a visible hypersensitive reaction developed at 72 h post-inoculation of super isolate CN152 on both attached and non-attached leaflets (data not shown). This reaction indicated that PTI was induced at an early infection of 24 h intervals, and ETI was induced at 48–72 h interval. A total of 3354 DEGs continuously contributed to different defense responses to late blight in SD20. In the first infection stage of 24 hpi, the DEGs mainly encoded defense enzymes and disease-related proteins, including osmotin, chitinase, glucan endo-1,3-beta-d-glucosidase, flavonoid 3-hydroxylase, and peroxidase. These genes were increased and activated by pathogens in early infection stages, thereby triggering a phosphorylation cascade through the mitogen-activated protein kinase (MAPK) signaling pathway that regulates downstream resistance-related protein genes, and finally to directly suppress the pathogen. 

As hpi increased, DEGs increased, especially the downregulated ones, which reached 1395 at 72 h, indicating that more and more downregulated genes need to be induced to generate resistance in the later stages of *Pi* infection. Interestingly, only four DEGS with the NBS-LRR domain were found in our study; the probable reason is that most resistance genes are constitutively expressed. Among the four NBS-LRR genes, one gene encoded homologues of tobacco mosaic virus (TMV) protein, and the other three genes were all homologous to possible disease-resistant proteins in potato. 

Transcription factors have been shown to play crucial roles in plant resistance [33]. Following pathogen attack, TFs regulated plant defense responses by modulating the transcription of downstream resistance related genes by binding to specific DNA sequences in their promoter regions [34,35]. The regulated resistance of numerous TFs belonging to families such as MYB, WRKY, NAC, bZIP, and ethylene responsive factor (ERF) have been proven in model plants [36,37]. However, relatively few members of TFs in potato defense against pathogens have been identified. Several transcription factors were also detected in our study including WRKY, bZIP, BHLH, MYB, and ERF families. Some have been reported in potato and other crops. Of these, *StWRKY1* was reported to regulate phenylpropanoid metabolites conferring late blight resistance in potato [38,39]. The β-aminobutyric acid (BABA) early induced *StWRKY5* gene was cloned from potato, and over expression of *StWRKY5* in potato enhanced late blight resistance [40]. Eleven WRKYs were strongly upregulated in this study, including WRKY1, WRKY3, and WRKY5, suggesting positive regulation of these WRKYs in the resistance response to *Pi* infection. 

Secondary cell wall biosynthesis, used to resist *Pi* infection, and its transcriptional regulation is mediated by NAC43 and MYB8 in potato [41]. Here, we found 17 MYB, 10 that were upregulated, including MYB4, MYB84, MYB161, and MYB164, and seven that were downregulated, including MYB6, MYB86, and MYB113. Overexpression of *TaMYB86* significantly increased resistance to common root rot in transgenic wheat lines [42]. In response to stress, plants produce TFs including ethylene responsive factors (ERFs). ERFs have a 58–59 amino acid DNA-binding domain called AP2/ERF that is conserved among plants. AP2/ERF modulates the expression of PR genes and GCC cis-elements by specifically binding to them. JA and ET trigger responses in ERF genes. In the present study, we also found six upregulated ERFs with AR2/ERF domains including TSRF1, ERF13 and ERF14. To regulate tolerance to salt and resistance to *Pi*, defense-related genes *PR1*, *NPR1*, and *WRKY1* are activated in potatoes along with *StERF3*, an ERF TF [43]. In the tomato, *PR* genes are activated by TSRF1, an ERF that binds to the GCC box allowing for positive regulation of pathogen resistance [44]. In tobacco, the osmotic response is negatively regulated by TSRF1, but pathogen resistance is still positively regulated [45]. Overexpression of *TSRF1* improved rice osmotic and drought tolerance [46]. Resistance to *Fusarium oxysporum*, a necrotrophic fungus, is mediated by *AtERF14*, which regulates expression of JA-responsive defense genes. *AtERF14* expression is required for expression of *ERF1* and *AtERF2*, genes involved in responding to ET/JA and in defense [47]. The future functional studies of these TFs induced in this study will be helpful for clarifying their specific roles in defense-related signaling pathways.

Specific molecules produced by pathogens or from damaged plant tissues are first recognized by receptor-like kinases (RLKs) existing at the plasma membrane in plants. RLKs primarily belong to the LRR class and positively regulate plant innate immunity [48,49]. In tomatoes, tobacco, and *Arabidopsis*, resistance against *Pi* requires the LRR-RLK SERK3/BAK1 [50,51]. The potato receptor-like kinase gene *StLRPK1* was involved in the response to late blight resistance [52]. In this study, we found 10 RLK genes, of which eight belonged to the LRR class and two were wall-associated kinases (WAKs). The two WAKs and one LRR-RLK gene were greatly upregulated at 24 hpi, which suggested that these RLKs are important pathogen-pattern recognition receptors at the early PTI stage in potato genotype SD20. 

### 3.4. Multi-Signaling Pathways Participated in Resistance and Defense against Pi in SD20

Preformed and induced responses are some of the mechanisms used by plants to defend against pathogens. Signaling pathways, most of which are regulated by salicylic acid (SA), jasmonic acid (JA), and ethylene (ET), are activated to make plants produce localized and systemic defenses by inducing expression of defense genes [53]. These defenses are used to resist biotrophic pathogens (mainly the SA-dependent pathway) and necrotrophic pathogens (mainly the JA and ET pathways, which typically regulate similar defense genes) [54,55].

In this study, GO enrichment analysis showed that the most significant enrichment of DEGs occurred with protein serine/threonine kinase activity, metabolic process, and biosynthetic process. The KEGG pathway analysis also demonstrated that the gene set was mostly located in metabolic pathways and biosynthesis of secondary metabolites. These results showed that several metabolic pathways are involved in the broad-resistance induced by super race CN152 in potato genotype SD20. Nineteen DEGs were enriched in HR reactions that encoded PAD4, MAPK, MKS1, receptor protein kinase, elicitor-responsive protein, and OAT. Some of these DEGs have been reported in other crops. These genes could play an important role in the broad-spectrum resistance to *Pi* infection in potato genotype SD20. *Arabidopsis* PAD4 functioned upstream from SA and was essential for defense against green peach aphid and the pathogens *Pseudomonas syringae* and *Hyaloperonospora arabidopsidis*. ETI in response to a virulent pathogen attack involved an acute local reaction in which PAD4 and its interacting partner ENHANCED DISEASE SUSCEPTIBILITY1 (EDS1) promoted a hypersensitive response characterized by cell death at infection sites in *Arabidopsis* [56]. MAPK genes of tobacco NtMEK2 and potato StMEK1 can all can activate the expression of SA-induced protein kinase (SIPK) and wound-induced protein kinase (WIPK), followed by the expression of resistance defense genes and explosion of reactive oxygen species, thereby conferring HR to late blight [57,58]. MAPK cascade also mediated the synthesis of plant antitoxins [59,60].

Among the 3354 annotated DEGs, we found key kinases and defense marker genes of different signaling pathways including PAL, PR-1, PR-2 and NDR1 for SA, LOX, AOS, and PR-3 for JA, and ACC for ET signaling pathways. *Arabidopsis* NDR1 and homologs of NDR1 increased disease resistance to pathogens in different plants [61]. Increased production of SA and expression of PR1 (a defense marker gene) led to this heightened resistance. Moreover, AP2/ERF transcription factors were involved in various signal transduction pathways, such as SA, JA, ET, and ABA, and were cross-talk factors in stress signaling pathways. We also found one gene coding for Small GTPases with 2-fold differential expression at 72 hpi. Zhang et al. (2014) [62] reported that in potato, resistance to *Pi* was negatively regulated by tobacco *AtROP1*, a small GTPases gene, due to accumulation of H_2_O_2_ mediated by NADPH oxidase; following this, expression of the *LOX* gene greatly increased. The further functional exploration of DEGs in this study indicated that multiple signaling pathways were associated with resistance defense to super race CN152 in tetraploid potato genotype SD20, including SA, JA, and ET pathways, and biosynthesis of secondary metabolites. 

In this study, a large number of DEGs were identified through transcriptome analysis. Despite the fact that true late blight resistance genes have not been identified, the results of this study provided an outline of the antiviral defense reaction and a deeper understanding of potato–oomycete interactions. Significantly, potato genotype SD20 is a well-characterized new source of resistance to *Pi*. Therefore, we will use multiple omics to investigate resistance genetic loci, combined with these transcriptome data, to speed up the separation and cloning of *R* genes in SD20, and then provide the resistant gene resources for potato late blight resistance breeding.

## 4. Materials and Methods

### 4.1. Plant Materials

In the previous study, we performed resistance identification to over 20 distinguished potato accessions and genotype SD20 with detached and non-detached leaves, and the result showed that only the potato genotype SD20 was highly resistant to the super race isolate CN152 and showed typical hypersensitive response (Figure 1).

Tissue cultured plants of SD20 were grown in glass bottles (72 × 59 mm) containing 30 mL MS medium supplemented with vitamins and 30 g·L^−1^ sucrose. Plants were cultivated at 24 °C under 16 h light/8 h dark for four weeks, with eight seedlings per bottle.

### 4.2. Inoculation with Phytophthora infestans Isolate

The *P. infestans* super race isolate CN152 (race 1, 3b, 4, 5, 6, 7, 8, 9, 10, 11) [13] was used in the present study. The isolate was activated 2–3 times on infected potato tuber slices and grown on rye agar medium supplemented with 2% sucrose for 7–14 days at 18 °C in closed Petri dishes to induce sporangia formation. Ice-cold tap water was added to the Petri dishes, followed by incubation for 3 h at 4 °C, to release zoospores from sporangia. The zoospore concentration was assessed by bright field microscopy using a Fuchs-Rosenthal counting chamber and adjusted to 5 × 10^4^ spores/mL. The spore suspension was sprayed with a hand sprayer onto the seedlings in each bottle, two bottles per sample were established as biological replicates, and H_2_O was used as a mock treatment. Seedlings were harvested at 0, 24, 48, and 72 h post-inoculation (hpi). Then, a total of 16 samples were frozen in liquid nitrogen immediately for RNA extraction and analysis. 

### 4.3. RNA-Seq Experiments

Total RNA was extracted using Trizol reagent (Invitrogen, Carlsbad, CA, USA), treated with TURBO DNase I (Ambion, Austin, TX, USA) for 30 min and purified using RNeasy^®^ Plant Mini Kit (QIAGEN, Hilden, Germany). RNA sequence libraries were prepared with TruSeq RNA sample Prep V2 kit (Illumina, San Diego, CA, USA) according to the manufacturer’s instructions. The quality and size of cDNA libraries were checked using Agilent 2200 TapeStation system (Agilent, Santa Clara, CA, USA) prior to sequencing. cDNA libraries were sequenced using the Illumina HiSeqX10 sequencing system with the 150-cycle paired-end sequencing protocol.

### 4.4. Analysis of RNA-Seq Datasets

Raw data in FASTQ format from the current trial are available from the Genome Sequence Archive (GSA) under accession CRA000806. Clean reads were obtained by removing reads containing adapters or Poly-N sequence as well as reads of low quality. All downstream analyses were based on high-quality, clean data. The reference accession, the doubled haploid *S. tuberosum* Group Phureja clone DM1-3 516R44 (hereafter referred to as DM) genome sequence (SolTub 3.0) and annotation files were downloaded from the ENSEMBL plants database (ftp://ftp.ensemblgenomes.org/pub/plants/release-34/fasta/solanum_tuberosum/dna/) [63]. HISAT2-build was used to build index files for the potato chromosome and scaffold sequences [22]. We used HISAT2 to align RNA-seq reads against the reference genome. The TUs and GTF-formatted files were assembled using cufflinks and cuffcompare [23]. Fragments per kilobase of exon per million fragments mapped of assembled transcripts (FPKM) were calculated and normalized using HTseq-count and DESeq2 [25,26].

### 4.5. Identification of Differentially Expressed Genes

We applied DESeq2 to normalize expression levels and perform differential expression analysis based on the negative binomial distribution [64]. The threshold of the *p*-value was determined by the false discovery rate (FDR). Genes with normalized expression fold-change greater than 2, and a *p*-value less than 0.05 were considered to be differentially expressed. The differentially expressed genes (DEGs) were annotated based on the functional annotation information of ENSEMBL release *Solanum tuberosum* SolTub_3.0 and the potato ortholog *Arabidopsis* genes.

### 4.6. Clustering Analysis

K-means clustering was performed by Euclidean distance method and each centroid was the mean of the points in that cluster. Hierarchical clustering of gene expression was performed by the clustergram function in R with default settings.

### 4.7. GO and KEGG Enrichment Analysis of Differentially Expressed Genes

Gene Ontology (GO) enrichment analysis of differentially expressed genes was implemented using agriGO (http://bioinfo.cau.edu.cn/agriGO/analysis.php) based on a hypergeometric test. KOBAS software (http://kobas.cbi.pku.edu.cn/index.php) was used to test for statistically significant enrichment of DEGs in KEGG (Kyoto Encyclopedia of Genes and Genomes) pathways. The ENSEMBL release *S. tuberosum* SolTub_3.0 was used as the entire GO term and KEGG population. GO and KEGG terms with a corrected *p*-value < 0.05 were considered significantly enriched for the DEGs. 

### 4.8. Validation of RNA-Seq Data by Real-Time Quantitative PCR (qRT-PCR)

Ten DEGs were randomly selected for qRT-PCR to verify the RNA-seq results. Primers for these 10 genes were designed using Primer 5 and are listed in Supplementary Table S2. The RNA samples were the same as the ones used for the RNA-seq analysis. A total of 1–2 μg of total RNA was used per 20 μL reaction for reverse transcription. PCR was performed in a 20-μL reaction mixture with 10 μL SYBR Premix *Ex Taq* (Takara, Japan), 0.5 μL of both forward and reverse primers, 7 μL of double-distilled H_2_O and 2 μL (40 ng/)μL of the cDNA. All qRT-PCR reactions were performed in triplicate for each cDNA sample with an annealing temperature of 60 °C and a total of 40 cycles of amplification. The expression level of each gene was calculated by the 2^−ΔΔ*C*t^ method using GAPDH as an internal reference [65].

## Figures and Tables

**Table 1:** Basic summary of sequence and sequencing reads mapping to the reference genome.

Sample	Total Reads	Total Bases (G)	Mapped Reads	Uniquely Mapped Reads
H_2_O_0h_rep1	100,379,690	14.73	85,471,167 (85.15%)	61,107,575 (60.88%)
H_2_O_0h_rep2	69,333,843	10.33	58,488,919 (84.36%)	40,952,418 (59.07%)
H_2_O_24h_rep1	72,144,160	10.70	58,778,306 (81.47%)	52,037,875 (72.13%)
H_2_O_24h_rep2	89,027,098	13.23	75,360,165 (84.65%)	54,980,812 (61.76%)
H_2_O_48h_rep1	70,254,604	11.67	59,645,988 (84.90%)	48,692,495 (69.31%)
H_2_O_48h_rep2	101,393,166	15.09	86,146,832 (84.96%)	59,650,502 (58.83%)
H_2_O_72h_rep1	88,635,452	13.11	72,436,689 (81.72%)	63,188,455 (71.29%)
H_2_O_72h_rep2	58,767,684	8.72	49,143,727 (83.62%)	37,364,283 (63.58%)
CN_0h_rep1	74,384,150	11.03	61,061,372 (82.09%)	51,846,675 (69.70%)
CN_0h_rep2	90,097,961	13.35	76,294,952 (84.68%)	53,857,487 (59.78%)
CN_24h_rep1	62,023,469	9.21	50,950,700 (82.15%)	42,735,432 (68.90%)
CN_24h_rep2	94,069,938	13.94	79,439,329 (84.45%)	55,514,195 (59.01%)
CN_48h_rep1	65,892,845	9.69	52,862,524 (80.22%)	44,911,407 (68.16%)
CN_48h_rep2	73,755,259	10.94	59,183,527 (80.24%)	43,261,168 (58.66%)
CN_72h_rep1	58,635,682	8.68	43,264,784 (73.79%)	36,679,517 (62.55%)
CN_72h_rep2	94,447,608	14.00	76,553,151 (81.05%)	54,108,598 (57.29%)

**Table 2:** The key enzymes and marker genes of SA, JA, and ET signaling pathways.

Gene ID	Description	log2FC_24 h	log2FC_48 h	log2FC_72 h
PGSC0003DMG400005492	Phenylalanine ammonia-lyase, PAL	0.56	2.72	3.26
PGSC0003DMG400023458	Phenylalanine ammonia-lyase, PAL	0.36	1.70	1.95
PGSC0003DMG402021564	Phenylalanine ammonia-lyase, PAL	0.93	1.99	2.24
PGSC0003DMG400019386	Phenylalanine ammonia-lyase, PAL	−0.37	1.03	1.22
PGSC0003DMG400005115	PR1 protein	0.00	1.94	0.71
PGSC0003DMG400005116	PR1 protein	−0.05	1.96	1.27
PGSC0003DMG400010635	BOP/NPR1/NIM1-like regulatory protein	−0.21	−0.54	−1.26
PGSC0003DMG400032231	NDR1	1.76	0.73	0.81
PGSC0003DMG400000519	Glucan endo-1,3-beta-glucosidase, acidic isoform GI9	4.47	3.49	4.23
PGSC0003DMG400029830	Glucan endo-1,3-beta-d-glucosidase	4.35	7.36	7.31
PGSC0003DMG400010491	Glucan endo-1,3-beta-d-glucosidase	2.99	3.89	5.50
PGSC0003DMG400018523	Glucan endo-1,3-beta-glucosidase	0.34	0.50	1.59
PGSC0003DMG400021848	Glucan endo-1,3-beta-glucosidase, basic vacuolar isoform GLB	0.03	1.81	1.81
PGSC0003DMG400012702	Glucan endo-1,3-beta-d-glucosidase	0.08	0.41	1.96
PGSC0003DMG400000689	Glucan endo-1,3-beta-glucosidase	−0.20	−0.73	−3.78
PGSC0003DMG400032133	Glucan endo-1,3-beta-glucosidase 4	−0.19	−0.65	−1.78
PGSC0003DMG400005021	Glucan endo-1,3-beta-glucosidase	−0.06	−0.73	−2.48
PGSC0003DMG402016475	Glucan endo-1,3-beta-glucosidase	−0.10	−0.61	−2.22
PGSC0003DMG400004264	Thaumatin	1.34	1.79	2.54
PGSC0003DMG400004262	Thaumatin	1.70	1.90	2.79
PGSC0003DMG400019873	Phytoalexin-deficient 4-2 protein, PDA4	1.63	2.46	2.93
PGSC0003DMG400010859	Lipoxygenase, LOX	4.15	7.88	9.08
PGSC0003DMG400024693	Lipoxygenase, LOX	1.65	0.42	0.45
PGSC0003DMG400032155	Lipoxygenase, LOX	1.45	0.71	−2.16
PGSC0003DMG400022894	Lipoxygenase, LOX	0.40	1.77	2.51
PGSC0003DMG400001149	Allene oxide synthase 2, AOS	1.30	1.46	1.25
PGSC0003DMG400010283	Class I chitinase	0.86	5.10	7.33
PGSC0003DMG400004593	Chitinase	−0.54	−1.03	−2.05
PGSC0003DMG400008673	Endochitinase (Chitinase)	4.47	4.92	5.83
PGSC0003DMG400001528	Class II chitinase	6.18	7.67	9.09
PGSC0003DMG400001529	Acidic 27 kDa endochitinase	1.71	1.86	1.72
PGSC0003DMG402001531	Chitinase 134	2.07	3.70	6.24
PGSC0003DMG400013894	1-aminocyclopropane-1-carboxylate oxidase 2, ACC	2.85	4.68	4.82
PGSC0003DMG400007999	Nbs-lrr resistance protein	1.71	2.37	2.70
PGSC0003DMG400027797	TIR-NBS-LRR disease resistance	0.72	0.80	1.10
PGSC0003DMG402004425	Cc-nbs-lrr resistance protein	−0.17	−0.59	−1.35
PGSC0003DMG400006800	NBS-LRR protein	−0.02	−0.79	−1.37

## Supplementary Materials

The following are available online at http://www.mdpi.com/1422-0067/19/6/1728/s1.
