# Supplementary material for: Gene Profiling in Late Blight Resistance in Potato Genotype SD20
Source: Int J Mol Sci. 2018 Jun 11;19(6):1728. doi: 10.3390/ijms19061728 (PMC6032139; doi:10.3390/ijms19061728)
Supplement: Supplementary file 1 [file ijms-19-01728-s001.pdf]

Table S1. Differential expressed genes involved in the immune response and HR actions with fold changes of their FPKM ratio (log2FC)

Annotated Gene	*Ath* ID	H_2O_0h FPKM	H_2O_24h FPKM	H_2O_48h FPKM	H_2O_72h FPKM	CN_0h FPKM	CN_24h FPKM	CN_48h FPKM	CN_72h FPKM	Description
PGSC0003DMG400007999	AT1G33560	18.0	50.4	37.4	40.7	20.1	67.9	122.9	143.8	Nbs-lrr resistance protein
PGSC0003DMG400000282	AT5G56760	2.8	3.6	2.9	2.8	2.8	3.9	6.2	7.1	Serine acetyltransferase 7
PGSC0003DMG400000321	AT3G24503	22.5	22.5	23.1	23.1	23.3	35.5	53.6	60.5	Aldehyde dehydrogenase
PGSC0003DMG400000422	AT2G27920	20.7	18.3	23.5	21.1	17.9	19.9	12.7	7.2	Retinoid-inducible serine carboxypeptidase
PGSC0003DMG400001096	AT5G54860	6.7	6.5	6.7	6.7	6.4	11.4	26.0	36.0	Transporter
PGSC0003DMG400001470	AT5G15730	6.1	7.0	6.6	6.8	6.1	9.6	15.0	14.1	Protein-serine/threonine kinase
PGSC0003DMG400003073	AT5G46420	20.3	14.6	18.1	21.4	19.2	12.0	9.2	5.3	16S rRNA processing protein RimM
PGSC0003DMG400004283	AT1G28680	16.2	20.8	18.7	17.7	15.8	36.1	31.9	41.7	EIG-I24 protein
PGSC0003DMG400004963	AT5G49660	31.5	38.3	36.6	29.0	35.1	30.7	22.1	12.5	Receptor protein kinase
PGSC0003DMG400004966	AT1G09970	9.3	21.0	16.3	15.9	9.2	25.2	53.7	58.8	Receptor protein kinase CLAVATA1
PGSC0003DMG400005407	AT3G06730	17.6	10.1	13.4	18.7	14.8	9.9	7.4	3.5	Thioredoxin-like protein CITRX
PGSC0003DMG400006084	AT5G58260	29.4	30.4	39.0	37.2	29.2	26.2	19.4	9.5	Conserved gene of unknown function
PGSC0003DMG400006800	NA	6.0	5.4	6.3	6.8	7.1	6.9	3.6	2.2	NBS-LRR protein
PGSC0003DMG400007993	AT2G26530	1.4	4.0	3.0	1.6	1.9	6.4	16.2	17.6	Pheromone receptor
PGSC0003DMG400008830	AT2G37660	127.1	96.5	115.0	130.9	114.8	87.3	67.0	27.5	NAD-dependent epimerase/dehydratase
PGSC0003DMG400008834	AT2G37630	58.9	45.6	46.9	64.0	58.7	46.7	29.3	7.2	Phantastica
PGSC0003DMG400009004	AT3G62600	29.2	29.7	25.6	28.1	26.8	49.5	81.4	87.5	Chaperone protein dnaJ
PGSC0003DMG400009238	NA	1.1	1.5	1.1	1.3	1.1	3.5	4.8	5.7	Leucine-rich repeat family protein
PGSC0003DMG400009317	AT1G32060	515.8	390.2	576.8	555.6	476.8	327.9	241.6	125.4	Phosphoribulokinase
PGSC0003DMG400009380	AT4G28490	10.3	11.7	11.4	10.3	10.8	11.3	8.1	3.7	Receptor protein kinase CLAVATA1
PGSC0003DMG400009883	AT1G76040	9.2	10.6	10.3	7.9	8.4	12.9	30.7	31.4	Calcium-dependent protein kinase
PGSC0003DMG400009913	AT1G42480	7.0	8.0	6.4	7.5	5.5	10.0	11.5	11.2	Conserved gene of unknown function
PGSC0003DMG400010870	AT2G17040	0.9	2.1	2.4	1.1	0.6	5.9	6.9	7.7	NAC domain protein
PGSC0003DMG400011274	AT1G16670	9.0	11.0	10.6	8.7	8.6	14.9	46.1	65.0	ATP binding protein
PGSC0003DMG400012396	NA	12.8	7.0	9.5	17.4	12.5	9.0	6.0	2.1	Lanceolate
PGSC0003DMG400012875	NA	76.4	86.6	71.4	71.1	67.9	133.9	163.5	197.8	Protein disulfide isomerase L-2
PGSC0003DMG400013751	AT4G03280	499.0	478.2	689.1	616.5	439.0	417.8	303.8	172.1	Cytochrome b6-f complex iron-sulfur subunit, chloroplastic

Annotated Gene	*Ath* ID	H_2O_0h FPKM	H_2O_24h FPKM	H_2O_48h FPKM	H_2O_72h FPKM	CN_0h FPKM	CN_24h FPKM	CN_48h FPKM	CN_72h FPKM	Description
PGSC0003DMG400014120	AT2G25110	55.5	64.5	56.0	57.2	54.0	84.7	119.6	127.9	Stromal cell-derived factor 2
PGSC0003DMG400016641	AT3G03300	8.4	6.2	6.5	7.7	8.1	6.3	4.4	2.4	ATP binding protein
PGSC0003DMG400016959	AT4G09650	360.1	303.0	437.5	384.5	300.5	259.3	193.4	89.1	ATP synthase delta chain, chloroplastic
PGSC0003DMG400017252	AT4G14465	3.4	7.5	7.1	5.3	4.8	5.8	10.5	10.7	DNA binding protein
PGSC0003DMG400019363	AT3G63190	107.2	86.6	104.7	120.4	93.8	67.2	56.3	20.6	Ribosome-recycling factor, chloroplastic
PGSC0003DMG400019873	AT3G52430	6.2	6.2	6.0	6.3	5.7	19.9	39.4	48.3	Phytoalexin-deficient 4-2 protein
PGSC0003DMG400025778	AT3G19370	2.2	3.1	2.8	2.0	2.3	5.9	12.9	13.9	Myosin heavy chain, fast skeletal muscle, embryonic
PGSC0003DMG400025778	AT3G19370	21.1	19.8	25.3	19.3	20.7	16.5	10.9	2.2	Myosin heavy chain, fast skeletal muscle, embryonic
PGSC0003DMG400026346	AT2G27310	3.8	52.2	8.6	4.1	7.5	19.9	61.3	93.5	F-box family protein
PGSC0003DMG400027125	AT3G55800	294.3	235.1	336.2	304.0	280.2	195.7	129.7	81.1	Chloroplast sedoheptulose-1,7-bisphosphatase
PGSC0003DMG400027523	AT5G04930	2.5	3.3	2.9	2.5	2.4	4.9	19.7	23.0	Phospholipid-transporting atpase
PGSC0003DMG400027742	AT3G55470	5.1	8.0	7.1	6.1	5.6	10.5	26.2	31.3	Elicitor-responsive protein
PGSC0003DMG400027797	NA	0.5	0.6	0.5	0.6	0.5	1.5	2.0	2.1	TIR-NBS-LRR disease resistance
PGSC0003DMG400028672	AT3G18690	3.9	5.3	3.9	3.8	3.8	7.0	11.5	10.8	Protein MKS1
PGSC0003DMG400029872	AT5G46180	8.2	9.6	9.5	8.3	7.9	11.1	18.0	38.7	Ornithine aminotransferase
PGSC0003DMG400030010	AT5G13190	21.2	27.6	23.4	24.2	21.9	34.9	65.0	88.4	Conserved gene of unknown function
PGSC0003DMG400030058	AT3G45640	13.0	23.3	17.5	16.7	13.6	44.4	117.5	164.6	MAP kinase
PGSC0003DMG400030612	NA	377.8	256.2	382.5	379.1	362.1	220.2	153.8	64.8	Serine hydroxymethyl-transferase, mitochondrial
PGSC0003DMG400030812	NA	144.1	176.6	158.3	170.5	147.5	341.8	925.7	956.0	Phospho-2-dehydro-3-deoxyheptonate aldolase 1, chloroplastic
PGSC0003DMG402004425	NA	12.6	10.2	11.8	12.7	12.7	11.2	8.5	4.4	Cc-nbs-lrr resistance protein

Ath, *Arabidopsis thaliana*; FPKM, Fragments per kilobase of exon per million fragments mapped; NA means non-significant orthologous in *Arabidopsis*.

Table S2. Primer sequences used for quantitative real-time PCR.

Gene	Forward Primer Sequence (5′-3′)	Reverse Primer Sequence (5′-3′)	Gene Description
PGSC0003DMG400026052	CAGGAAACAGGGCAACTC	ATCACGGCTGGCATAACT	AvrPto-dependent Pto-interacting protein 3
PGSC0003DMG400030364	AACCCTTACCCTAACTCACT	CAGAATATCCATCACCCTCA	Avr9/Cf-9 rapidly elicited protein 284
PGSC0003DMG400015342	ATGTTTATGCCCATCCTACT	TTGATTTCTGTTCCCGCT	Jasmonic acid 2
PGSC0003DMG400025481	ACTTCGTCGGAGAATGGA	GTGGGATGTCTGACAACTTC	WRKY domain class transcription factor
PGSC0003DMG400034322	TTATTCAAGTGTAGCGAGTGTC	CCCAAAGCCTGTCCAGTT	Zinc finger protein
PGSC0003DMG400032555	CGAATCATCGGAGTCAAT	TTCTGTGGTCTTCGTCCC	NAC domain protein
PGSC0003DMG400006369	CGTATGAACAATCGCAATCT	AGCCCATGATCCTGAACTC	AP2/ERF domain-containing transcription factor
PGSC0003DMG400007999	TTATGCCGTTCAAGTGCC	ATCTCGGAGAAGTTTGGAG	Nbs-lrr resistance protein
PGSC0003DMG400030058	ATTATCACGATGCCAACC	GAAAGCACCCAAGTCCAG	MAP kinase
PGSC0003DMG400003043	ACGCAACAACTGTCCATAC	AGACTCCACCACAATCACC	Osmotin
GAPDH	CTGGTGCTGACTTCGTCG	CTGGCTTGTATTCATTCTCG	Internal reference gene
